# Bayesian updating: increasing sample size during the course of a study

**DOI:** 10.1186/s12874-021-01334-6

**Published:** 2021-07-05

**Authors:** Mirjam Moerbeek

**Affiliations:** Department of Methodology and Statistics, Utrecht University, PO Box 80140, 3508 TC Utrecht, the Netherlands

**Keywords:** Bayes factor, Informative hypothesis testing, Error rate

## Abstract

**Background:**

A priori sample size calculation requires an a priori estimate of the size of the effect. An incorrect estimate may result in a sample size that is too low to detect effects or that is unnecessarily high. An alternative to a priori sample size calculation is Bayesian updating, a procedure that allows increasing sample size during the course of a study until sufficient support for a hypothesis is achieved. This procedure does not require and a priori estimate of the effect size. This paper introduces Bayesian updating to researchers in the biomedical field and presents a simulation study that gives insight in sample sizes that may be expected for two-group comparisons.

**Methods:**

Bayesian updating uses the Bayes factor, which quantifies the degree of support for a hypothesis versus another one given the data. It can be re-calculated each time new subjects are added, without the need to correct for multiple interim analyses. A simulation study was conducted to study what sample size may be expected and how large the error rate is, that is, how often the Bayes factor shows most support for the hypothesis that was not used to generate the data.

**Results:**

The results of the simulation study are presented in a Shiny app and summarized in this paper. Lower sample size is expected when the effect size is larger and the required degree of support is lower. However, larger error rates may be observed when a low degree of support is required and/or when the sample size at the start of the study is small. Furthermore, it may occur sufficient support for neither hypothesis is achieved when the sample size is bounded by a maximum.

**Conclusions:**

Bayesian updating is a useful alternative to a priori sample size calculation, especially so in studies where additional subjects can be recruited easily and data become available in a limited amount of time. The results of the simulation study show how large a sample size can be expected and how large the error rate is.

**Supplementary Information:**

The online version contains supplementary material available at 10.1186/s12874-021-01334-6.

## Introduction

One of the main questions in the design phase of an empirical study is how large the sample size should be. The answer to this question is often found by means of a statistical power analysis [1, 2]. If an effect exists in the population, then a researcher should be able to find it with sufficient probability. This probability is known as the statistical power and it can be shown to be related to sample size, effect size and type I error rate. Nowadays, many software packages are available to facilitate a power analysis, such as G*power [3, 4], nQuery Advisor [5], and PASS [6]. However, it is not always easy to perform a power analysis because power is a function of effect size, of which the value is often not known in the design phase of a study. This causes a vicious cycle: the aim of a study is to gain insight in the size of the effect, but to plan the sample size of a study the size of the effect must be known beforehand. It is often advocated to escape this vicious cycle by using an a priori estimate that is based on expert knowledge or expectations, or findings in the literature. However, there is no guarantee such an a priori estimate is correct. An estimate that is too large results into too small a sample size and hence a risk of not finding a significant effect. On the other hand, an estimate that is too small results in too large a sample size and hence is a waste of resources.

Instead of performing an a priori sample size calculation, it is also possible to re-estimate sample size during the course of a study. Some pilot data can be collected and used to estimate model parameters, such as the effect size and residual variances, which in their turn can be used to calculate the required sample size to achieve a user-specified power level. Stein [7] was the first to propose sample size re-estimation. He argued that the power for a two-group comparison depends on the variance. He proposed to first sample some pilot data to be used to calculate the variance. Based on these pilot data, the required sample size is calculated and a second sample of this size is drawn.The pilot data are not used in the final analysis, hence the type I error rate is preserved. Wittes and Brittain [8] proposed an adjustment that uses all data, including the pilot data, in the final analysis. Based on the parameter estimates from the pilot data it is estimated how large the sample size should be to achieve sufficient power. If this sample size is larger than the size of the pilot, then additional data are collected. Otherwise, data collection is terminated. In this approach the data are treated as if the two phases of data collection are independent, while in fact they are not hence the type I error rate $\alpha$ may not always be preserved [8, 9].

In group sequential trials the sample size may be adjusted more than once [10, 11]. Before data collection, it has to be determined how often an interim analysis is done and how many additional subjects are to be collected between each pair of adjacent interim analyses. The $\alpha$-level at each interim test is chosen such that the overall $\alpha$-level is preserved. For each interim test the value of the test statistic is calculated based on the data collected thus far. If this test statistic exceeds a boundary value, which is determined based on the $\alpha$-level at that interim test, then no further data are collected. Otherwise, data collection continues until the next interim test. It may occur the test statistic at the final test does not exceed the boundary value. In that case it is not allowed to collect further data since all type I error has already been spent. This may be considered a drawback of the group sequential trial design. It is therefore important to weight the risks of an inflated type I error in a group sequential design against the risk of an under- of overpowered study when using an a priori sample size calculation while making a choice among these two procedures of sample size determination.

There exists another procedure for increasing sample size during the course of a study: Bayesian updating. This procedure does not depend on the Neyman-Pearson approach of null-hypothesis significance testing [12], but uses another approach based on the Bayes factor [13, 14]. The Bayes factor quantifies the support in the data for an informative hypothesis, and can also be used to quantify the relative support of two competing informative hypothesis. Such informative hypotheses are based on subjective beliefs, expectations or findings in the literature. Recent research has focussed on a priori sample size calculations for informative hypothesis testing [15]. Again, sample size depends on the effect size, hence we end up in the same vicious cycle as described previously. However, it is possible to increase the sample size during the course of the study until sufficient support for a hypothesis is achieved, without making a decision upfront about the number of times sample size will be increased. In addition, since the Bayesian approach does not use a test statistic and type I error rate, there is no need to decide about how the $\alpha$-level should be adjusted each time the sample size is increased. This makes Bayesian updating a much more flexible approach than group sequential trials.

In recent years Bayesian updating has received attention in the social and behavioural science literature [16–19]. The aim of the current paper is to introduce Bayesian updating to researchers in the biomedical field. This paper consists of two parts. The first explains how informative hypotheses can be tested by using the Bayes factor, and how the Bayes factor is used in Bayesian updating. The second part presents a simulation study that evaluates Bayesian updating in two-group comparisons. The results of this simulation study give insight in what sample sizes can be expected in Bayesian updating and how large the error rate is. An error occurs when the data show most support for the incorrect hypothesis, that is, the hypothesis that was not used to generate the data.

The simulation study extends previous simulation studies on Bayesian updating for two-group comparisons [17, 18]. It does not only focus on the t-test for equal variances but also for unequal variances. The latter is also known as Welch’s test. Furthermore, it uses three sets of two competing hypotheses rather than just one such a set. In addition to that, it explores the effects of the group size at the beginning of the study, and the consequences of using a maximum group size. Finally it uses a different approach to calculate the Bayes factor. This approach is known as the Approximate Adjusted Fractional Bayes factor (AAFBF) approach [20, 21]. With this approach a fraction parameter must be specified to control the amount of information in the dat used to specify a prior. The remainder of information is used to test informative hypotheses. This approach will be explained in the next section.

## Informative hypothesis testing using the Bayes factor

Informative hypotheses are formulated on the basis of a researcher’s beliefs, expectations, or findings in the literature, and do not necessarily have to include the null hypothesis. Consider as an example a trial in which two pain killers A and B are compared to a placebo. The response variable measures the level of pain; the higher the score, the more pain the respondent experiences. In the framework of null hypothesis significance testing one would formulate the null hypothesis ${H}_{0}:{\mu }_{A}={\mu }_{B}={\mu }_{P}$, where ${\mu }_{A}$, ${\mu }_{B}$ and ${\mu }_{P}$ are the mean scores for pain killers A and B and the placebo, respectively. However, researchers often do not believe such a null hypothesis of equal group means to be true and will use equality and inequality constraints on the three group means to formulate informative hypotheses. For instance, the manufacturer of pain killer A may believe its pain killer to be most effective and pain killer B to be more effective than the placebo, resulting in the following informative hypothesis ${H}_{1}:{\mu }_{A}<{\mu }_{B}<{\mu }_{P}$. The manufacturer of pain killer B may come up with the following competing informative hypothesis ${H}_{2}:{\mu }_{B}<{\mu }_{A}<{\mu }_{P}$. Finally, a consumer may believe both pain killers to be more effective than the placebo, which results in informative hypothesis ${H}_{3}:({\mu }_{A},{\mu }_{B})<{\mu }_{P}$, where the comma between the two means ${\mu }_{A}$ and ${\mu }_{B}$ implies no constraint is placed on these two means. In a similar manner, informative hypothesis can be formulated for other types of statistical models, such as in regression models (e.g. comparing the effects of father’s and mother’s educational levels on their child’s weight) and in mediation models (comparing direct and indirect effects).

Informative hypotheses can be tested by means of the Bayes factor. The Bayes factor ${BF}_{iu}$ of informative hypothesis ${H}_{i}$ versus the unconstrained hypothesis ${H}_{u}=\boldsymbol{\mu }$ is expressed in a simple form: ${BF}_{iu}={f}_{i}/{c}_{i}$. The unconstrained hypothesis is simply the parameter space: all possible combinations of the values of the three group means ${\mu }_{A}$, ${\mu }_{B}$ and ${\mu }_{P}$. The complexity ${c}_{i}\in [\mathrm{0,1}]$ is the proportion of the prior distribution that is in agreement with the hypothesis ${H}_{i}$. The lower its value, the more parsimonious hypothesis ${H}_{i}$ is. The fit ${f}_{i}\in [\mathrm{0,1}]$ is the proportion of the posterior distribution that is in agreement with the hypothesis ${H}_{i}$.

**Fig. 1:**
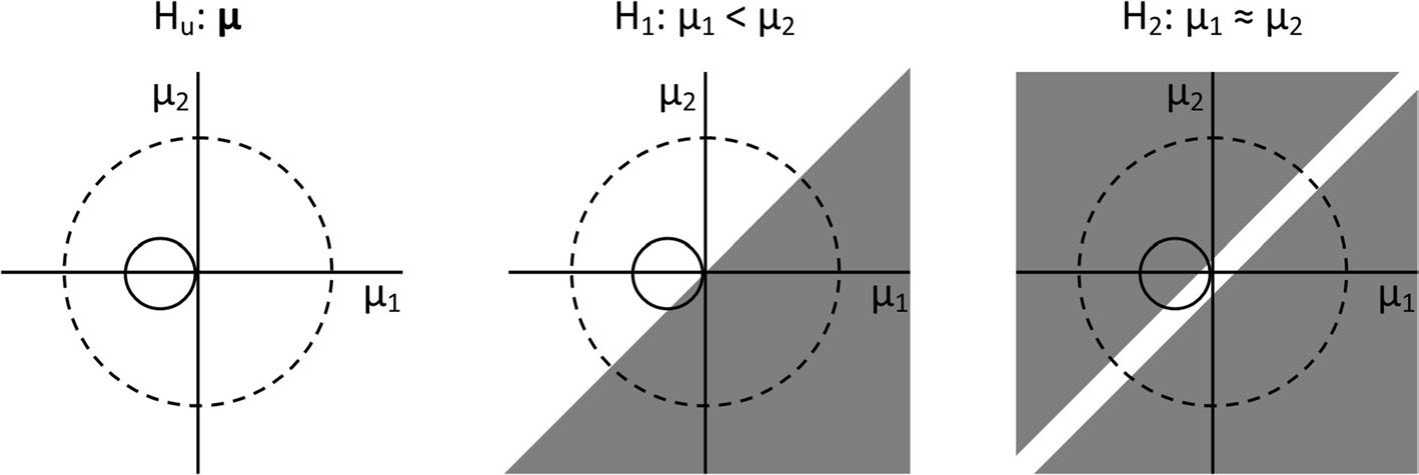
Prior and posterior distributions for two-group comparisons with three different informative hypotheses

Figure 1 gives a representation of fit and complexity for a two-group comparison on a quantitative response variable. The three panels give a two-dimensional presentation of the prior (dashed circle) and posterior (solid circle) of two independent means ${\mu }_{1}$ and ${\mu }_{2}$. The panel at the left uses the unconstrained hypothesis ${H}_{u}:\boldsymbol{\mu }$. This hypothesis does not put any equality or inequality constraints on the two means. In other words, it implies that anything can be going on with respect to these two means. The complexity of this hypotheses is the proportion of the prior (i.e. the area within the dashed circle) that is in agreement with the hypothesis; by default it has the value 1 for the unconstrained hypothesis. Similarly, the fit is the proportion of the posterior (i.e. the area within the solid circle) that is in agreement with the hypothesis; by default it also has the value 1 for the unconstrained hypothesis. The panel in the middle uses the inequality constrained hypothesis ${H}_{1}:{\mu }_{1}<{\mu }_{2}$. Only those parts of the prior and posterior that are not overlapped by the grey triangle are in agreement with the hypothesis. It can be seen the complexity is one half and the fit is a little less than one. Hypothesis ${H}_{1}$ is more parsimonious than hypothesis ${H}_{u}$ since it has a lower complexity. The panel at the right uses an approximate equality ${H}_{2}:{\mu }_{1}\approx {\mu }_{2}$. The parts of the prior and posterior that are in agreement with the hypothesis (i.e. the areas of the two circles that are not overlapped by the two grey triangles) are even smaller than in the middle panel, implying an even lower complexity and fit. This hypothesis is hence the most parsimonious of the three.

The statistical model is a linear model for a continuous outcomes and two groups. The continuous outcome ${y}_{i}$ of subject $i = 1, \ldots ,2n$ is given by:

$${y}_{i}={\mu }_{1}{D}_{1i}+{\mu }_{2}{D}_{2i}+{\varepsilon }_{i},$$

where ${\mu }_{1}$ and ${\mu }_{2}$ are the means in groups 1 and 2, respectively. ${D}_{1i}=1$ if subject $i$ is in group 1 and 0 otherwise, and ${D}_{2i}=1$ if subject $i$ is in group 2 and 0 otherwise. The residual follows a normal distribution. In the case of equal within-group variances of the two groups: ${\varepsilon }_{i}\sim N(0,{\sigma }^{2})$. In the case of unequal within-group variances of the two groups: ${\varepsilon }_{i}\sim N(0,{D}_{1i}{\sigma }_{1}^{2}+{D}_{2i}{\sigma }_{2}^{2})$.

The prior distribution of $\boldsymbol{\mu }=({\mu }_{1},{\mu }_{2})$ is based on the fractional Bayes factor approach [22, 23] and is constructed by using a fraction of information in the data $y$. In other words, the user does not have to specify a distribution for the prior. For the case of an unequal variances t-test

$$h\left(\boldsymbol{\mu }|{y}_{1}\right)=N\left(\left[\begin{array}{c}0\\ 0\end{array}\right],\left[\begin{array}{cc}\frac{1}{b}\frac{{\widehat{\sigma }}_{1}^{2}}{n}& 0\\ 0& \frac{1}{b}\frac{{\widehat{\sigma }}_{2}^{2}}{n}\end{array}\right]\right),$$

where ${y}_{1}$ are the data to construct the prior. The prior is a bivariate normal distribution with $n$ the sample size per group and ${\widehat{\sigma }}_{1}^{2}$ and ${\widehat{\sigma }}_{2}^{2}$ the unbiased estimates of the within-group variances. In the case of equal variances, ${\widehat{\sigma }}_{1}^{2}$ and ${\widehat{\sigma }}_{2}^{2}$ are replaced by ${\widehat{\sigma }}^{2}$. Furthermore, $b$ is the fraction in the data used to specify the prior distribution. The default value of $b$ is $\frac{1}{2n}$, and this choice is inspired by the minimal training sample [24, 25] so that an noninformative prior is turned into a proper prior by using a small amount of information in the data via a normal approximation. This value implies half a subject is taken from each group, so one subject in total.

It should be noted that, as the two group means are zero, the prior distribution is not used to represent prior knowledge about the effect size under any informative hypothesis (i.e. the solid circle in Fig. 1 is centred around the origin $\left({\mu }_{1},{\mu }_{2}\right)=(\mathrm{0,0})$). In other words, subjective input from the researcher is not needed to specify the prior. However, it is needed to specify informative hypotheses by using equality and inequality constraints on the group means.

The posterior distribution of $\boldsymbol{\mu }=({\mu }_{1},{\mu }_{2})$ is a bivariate normal approximation given by

$$g\left(\boldsymbol{\mu }|{y}_{2}\right)=N\left(\left[\begin{array}{c}{\widehat{\mu }}_{1}\\ {\widehat{\mu }}_{2}\end{array}\right],\left[\begin{array}{cc}\frac{{\widehat{\sigma }}_{1}^{2}}{n}& 0\\ 0& \frac{{\widehat{\sigma }}_{2}^{2}}{n}\end{array}\right]\right),$$

where ${\widehat{\mu }}_{1}$ and ${\widehat{\mu }}_{2}$ are the maximum likelihood estimates of the two group means. These means may be different from zero, hence the dashed circle in Fig. 1 is not necessarily centred around the origin. This equation holds for the case of unequal variances; in the case of equal variances, ${\widehat{\sigma }}_{1}^{2}$ and ${\widehat{\sigma }}_{2}^{2}$ are replaced by ${\widehat{\sigma }}^{2}$. It should be noted that the posterior is constructed from the prior and ${y}_{2}$, which is the part of the data that is not used to construct the prior.

The Bayes factor ${BF}_{iu}$ quantifies the support for a hypothesis ${H}_{i}$ versus the unconstrained hypothesis ${H}_{u}$. It is also possible to calculate the relative support of a hypothesis ${H}_{a}$ versus another hypothesis ${H}_{b}$: ${BF}_{ab}={BF}_{au}/{BF}_{bu}$. If ${BF}_{ab}=1$ then both hypotheses receive equal support from the data; if ${BF}_{ab}>1$ then ${H}_{a}$ receives most support from the data and if ${BF}_{ab}<1$ then ${H}_{b}$ receives most support from the data. There exist various guidelines in the literature for the interpretation of the value of ${BF}_{ab}$. Table 1 repeats the classification scheme that has been published earlier in this journal [26]. It should be mentioned that this scheme should not be used in a stringent manner, such as the type I error rate $\alpha$ is used to distinguish significant and insignificant effects in null hypothesis significance testing. Some Bayesian statisticians even recommend not using such schemes at all, but only reporting the value of the Bayes factor such that the reader can make his or her own judgment.

**Table 1:** Classification scheme for the Bayes factor ${BF}_{ab}$

${BF}_{ab}$	Interpretation
>100	Extreme support for ${H}_{a}$
30-100	Very strong support for ${H}_{a}$
10-30	Strong support for ${H}_{a}$
3-10	Moderate support for ${H}_{a}$
1-3	Anecdotal support for ${H}_{a}$
1	Support for neither hypothesis
1/3-1	Anecdotal support for ${H}_{b}$
1/10-1/3	Moderate support for ${H}_{b}$
1/10-1/30	Strong support for ${H}_{b}$
1/30-1/100	Very strong support for ${H}_{b}$
<1/100	Extreme support for ${H}_{b}$

## Illustrative example: comparing cholesterol levels across males and females

The publicly available Framingham dataset [27] contains physiological measurements from 669 males and 737 females. In this illustration males and females are compared with respect to their serum cholesterol levels (measured in mg/100 ml). For illustrative purposes, a random sample of only 100 males and 100 females from this data set is used. Two informative hypotheses are compared: ${H}_{0}:{\mu }_{males}={\mu }_{females}$ and ${H}_{1}:{\mu }_{males}<{\mu }_{females}$.

Table 2 presents results for the two parameters ${\mu }_{males}$ and ${\mu }_{females}$ that are used to specify the informative hypotheses: the estimate, the standard deviation of the posterior distribution and the 95% credible interval. The latter is the interval bounded by the 2.5 and 97.5% quantiles of the posterior distribution. The estimate for females is larger than the estimate for males and the credible intervals overlap somewhat.

**Table 2:** Summary statistics for the Framingham example

Parameter	Estimate	Posterior s.d.	95% credible interval
Mean for males	223.04	5.69	(211.9, 234.2)
Mean for females	247.00	9.09	(229.2, 264.8)

Table 3 shows the fit, complexity, and Bayes factor for both hypotheses. The fit and complexity for hypothesis ${H}_{0}$ are very small and the Bayes factor ${BF}_{0u}={f}_{i}/{c}_{i}=0.987/0.5=0.824$ shows there is more support for hypothesis ${H}_{u}$ than for ${H}_{0}$ in the data. The fit for hypothesis ${H}_{1}$ is equal to 0.987, so 98.7% of the posterior is in agreement with the hypothesis. The complexity is 0.5, so 50% of the prior is in agreement with the hypothesis. ${BF}_{1u}=1.975$ meaning the support in the data for ${H}_{1}$ is almost twice as large as for ${H}_{u}$. The ratio of the two Bayes factors is ${BF}_{10}={BF}_{1u}$/${BF}_{0u}=1.975/0.824=2.40$, which implies hypothesis ${H}_{1}$ receives 2.40 times as much support from the data than hypothesis ${H}_{0}$. Such an amount of support is considered anecdotal (see Table 1).

**Table 3:** Fit, complexity and Bayes factor for the two hypotheses of the Framingham example

Hypothesis ${H}_{i}$	Fit ${f}_{i}$	Complexity ${c}_{i}$	Bayes factor ${BF}_{iu}$
${H}_{0}:{\mu }_{males}={\mu }_{females}$	0.003	0.004	0.824
${H}_{1}:{\mu }_{males}<{\mu }_{females}$	0.987	0.5	1.975

## Bayesian updating

The Framingham dataset is used to illustrate Bayesian updating. Suppose one aims for a strong amount of support for either hypothesis ${H}_{0}$ or ${H}_{1}$ from the data. First, 20 subjects per gender are used to calculate the Bayes factor. Subsequently, the sample size per gender is increased by adding one subject and the Bayes factor is calculated again. This is done until strong support for one of the two hypotheses is found: the Bayes factor ${BF}_{10}$ exceeds the target value ${BF}_{target}=10$ (which implies more support for ${H}_{1}$) or subceeds its complement ${1/BF}_{target}=1/10$ (which implies more support for ${H}_{0}$).

**Fig. 2:**
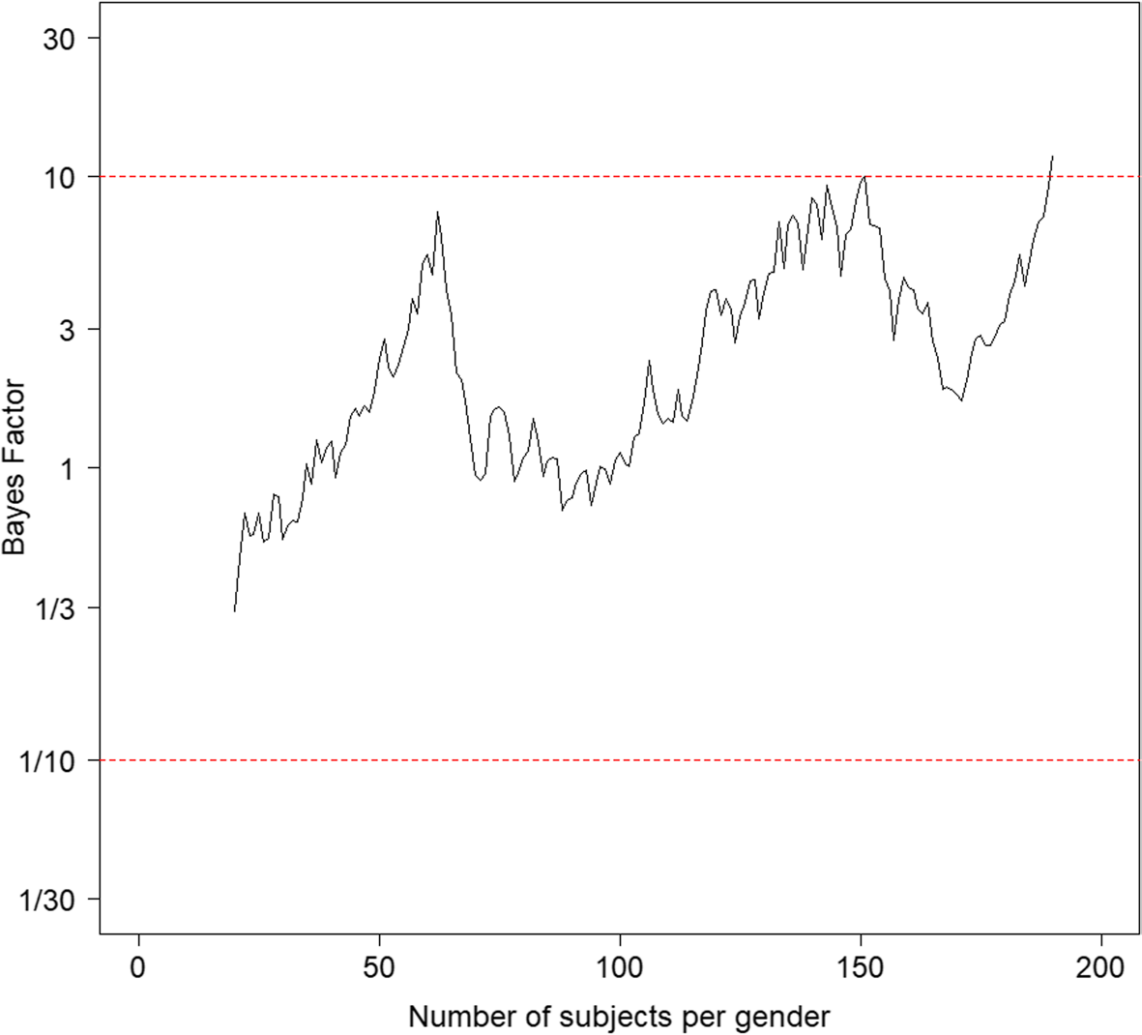
Example of a trajectory in Bayesian updating: comparison of cholesterol levels between males and females

Figure 2 shows the Bayes factor as a function of the number of subjects per gender. The two horizontal dashed lines are the target value and its inverse. For small number of subjects per gender the data show more support for ${H}_{0}$ than for ${H}_{1}$. As sample size increases, the support for ${H}_{1}$ becomes stronger and almost sufficient support is achieved for 62 subjects per gender. However, the Bayes factor decreases to lower values if the sample size further increases to 72. This is because during this period of data collection the males had a (much) larger serum cholesterol level than the females. Only after 100 subjects per gender are included a steady increase of the Bayes Factor is observed. Once 190 subjects per gender are included the boundary ${BF}_{target}=10$ is exceeded and the process of adding subjects terminates. Most support is then found for ${H}_{1}$ as the Bayes factor is equal to 11.7.

Various adjustments to the procedure described above are available. First, the number of subjects to be added in each step may be larger than just one and it may even change during the course of the study. For instance, in a trial that compares treatments for a rare disease or condition, recruiting subjects may be relatively easy at the beginning of the study but may become more difficult later on. It is also possible the number of added subjects is different across the two groups. Second, the initial sample size per group may be smaller or larger than 20. With a large initial sample size sufficient support for one hypothesis may be found immediately, meaning the duration of the study may be short. However, in such a case the sample size may be larger than actually needed. In other words, sufficient support for either hypothesis could have been achieved with a smaller group size. This may be problematic in trials in which recruiting, treating and measuring subjects is expensive and/or when treatments have harmful side-effects. On the other hand, using a small initial sample size may result in the incorrect hypothesis getting most support from the data due to chance. Third, there may be a limit on the sample size, which implies it is possible neither hypothesis gets a sufficient amount of support from the data once the maximum sample size is reached. In other words, the Bayes factor does not exceed ${BF}_{target}$ or subceed its inverse $1/{BF}_{target}$. The likelihood of such an inconclusive result is likely to increase with decreasing effect size and increasing ${BF}_{target}$.

## Simulation study for two-group comparisons

### Design of simulation study

A simulation study was conducted to answer three questions on Bayesian updating in two-group comparisons:

1. What sample sizes can be expected?
2. How large are the error rates: how often does the Bayes factor show more support for the hypothesis that was not used to generate the data?
3. In the case the sample size per group is limited to a certain maximum: how often is the result inconclusive?

The simulation study included seven factors. These factors and their chosen levels are as follows:

1. The set of two hypotheses to be compared. Three sets are considered. The first set compares the null hypothesis of equal group means ${H}_{0}:{\mu }_{1}={\mu }_{2}$ to a one-sided alternative hypotheses ${H}_{1}:{\mu }_{1}<{\mu }_{2}$. The second compares the same null hypotheses to a two-sided alternative hypothesis ${H}_{1}:{\mu }_{1}\ne {\mu }_{2}$. The third compares two one-sided hypotheses to each other: ${H}_{1}:{\mu }_{1}>{\mu }_{2}$ and ${H}_{2}:{\mu }_{1}<{\mu }_{2}$.
2. The effect size, for which four different values are considered: Cohen’s $d=0$, 0.2, 0.5 and 0.8. These reflect zero, small, medium and large effects. A zero effect size is not used for those scenarios that use the third hypotheses set.
3. The target BF, for which four different values are considered: ${BF}_{target}=3$, 5, 10 and 20. Sufficient support for the first hypotheses in each of the three hypotheses sets is achieved when ${BF>BF}_{target}$ and sufficient support for the second hypothesis is achieved when ${BF<1/BF}_{target}$.
4. The fraction $b$ in the data used to specify the prior distribution. Three different values are used: *b*, *2b* and *3b*, meaning that one, two and three subjects are used in total to specify the prior.
5. The type of test. With the equal variances t-test the variances in both groups are equal and in the simulation ${var}_{1}={var}_{2}=1$ was used. With the unequal variances t-test (i.e. Welch’s test) unequal variances are considered in both group and data were simulated with ${var}_{1}=4/3$ and ${var}_{2}=2/3$ (i.e. the average variance is 1, just as the variance for the t-test).
6. The minimum group size: the number of subjects per group at the start of the study. This is the group size for which the Bayes Factor is calculated for the first time. Three different values are used: ${N}_{min}=5$, 10 and 20.
7. The maximum group size: the maximum number of subjects that can be recruited per group. Four different values are used: ${N}_{max}=5$ 0, 100, 200 and 50,000. The latter serves as a proxy for an unlimited group size.

In total 3168 combinations of factor levels were considered in this simulation study; these are called scenarios in the remainder of this contribution. Note that this is not a full factorial design since for the third hypothesis set Cohen’s $d=0$ cannot be considered. For each of those 5,000 replications were generated, which gives a total of 15,840,000 replications. To keep the simulation manageable, the step size (i.e. the number of subjects added to each group before the Bayes Factor is calculated again) for increasing group size depended on the group size $N$. For $N<100$ the step size was 1, for $100<N<100$ 0 the step size was 5, for $1000<N<250$ 0 the step size was 10, for 25 $00<N<500$ 0 the step size was 20 and for 5 $000<N<5000$ 0 the step size was 50. All data were generated in R, version 4.0.2 [28]. For each data set the R function t.test with either equal or unequal variances was used. Subsequently, calculation of Bayes factors was done using the same version of R and the R package bain [16, 20].

The output for each scenario consists of two elements. The first is the distribution of the group size $N$ at which $BF$ exceeds the threshold ${BF}_{target}$ or subceeds its inverse ${1/BF}_{target}$, or the maximum group size is achieved. The second is the distribution of the corresponding value of $BF$. From the latter is can be derived how often the incorrect hypothesis gets most support from the data, and how often the result is inconclusive.

### Results of simulation study

The results of the simulation study can be explored in a Shiny app that is available at https://utrecht-university.shinyapps.io/BayesianUpdating/. This Shiny app allows the user to study the distribution of $N$ and $BF$ for any combination of factor levels that were used in the simulation study. Furthermore, it also gives the mean, median and maximum group size, and the percentage data sets for which the correct hypothesis, the incorrect hypothesis or neither hypothesis is favoured (i.e. an inconclusive result).

This section discusses some general findings. Table 4 shows the error rates and mean group size as a function of the hypotheses set, effect size, the fraction in the data used to specify the prior and the target $BF$. The results in this table hold for a t-test, with a minimum group size of 20 and a maximum group size of 50,000. This group size served as a proxy for an unlimited group size. Only in 2 out of the 660,000 replications this maximum group size was reached, which is a negligible amount.

**Table 4:** Percentage error and mean sample size for the equal variances t-test $\left( {N_{{min}} = 20,N_{{max}} = 50000} \right)$

			BF_target_ = 3		BF_target_ = 5		BF_target_ = 10		BF_target_ = 20	
HypSet	ES	Fraction	% error	Mean N	% error	Mean N	% error	Mean N	% error	Mean N
1	0	1b	5.1	22	4.1	29	2.5	70	1.5	296
1	0	2b	7.2	24	5.7	36	3.7	123	2.3	523
1	0	3b	9.1	26	7.1	47	4.4	179	3.1	799
1	0.2	1b	78.9	25	72.1	49	47.0	171	13.6	406
1	0.2	2b	70.8	29	61.3	69	27.3	238	3.6	451
1	0.2	3b	65.2	32	51.3	79	19.0	268	1.5	435
1	0.5	1b	35.0	26	19.9	37	4.3	58	0.2	74
1	0.5	2b	24.7	27	10.7	39	0.8	56	0.0	68
1	0.5	3b	17.9	28	6.5	40	0.2	54	0.0	63
1	0.8	1b	6.1	22	1.9	25	0.1	28	0.0	31
1	0.8	2b	3.2	22	1.0	24	0.0	27	0.0	30
1	0.8	3b	1.8	22	0.5	24	0.0	26	0.0	29
2	0	1b	5.0	22	3.9	27	3.8	92	2.1	380
2	0	2b	7.4	23	6.3	42	4.2	180	2.8	758
2	0	3b	9.8	26	7.7	62	5.3	265	3.5	1114
2	0.2	1b	88.3	23	84.7	34	56.6	170	10.1	512
2	0.2	2b	82.4	25	73.3	56	30.0	282	1.1	543
2	0.2	3b	78.9	28	62.2	83	17.2	342	0.0	531
2	0.5	1b	50.5	24	34.7	35	2.5	70	0.0	85
2	0.5	2b	39.8	26	15.4	44	0.1	67	0.0	78
2	0.5	3b	31.9	28	6.3	48	0.0	64	0.0	75
2	0.8	1b	13.9	23	4.7	26	0.0	31	0.0	34
2	0.8	2b	7.7	23	1.1	26	0.0	29	0.0	33
2	0.8	3b	5.6	23	0.1	26	0.0	29	0.0	32
3	0.2	1b	19.2	26	12.8	41	7.0	71	3.8	114
3	0.2	2b	18.5	26	13.1	40	7.3	71	3.3	114
3	0.2	3b	19.8	26	13.3	39	7.4	70	3.4	113
3	0.5	1b	2.1	21	0.8	23	0.3	26	0.2	31
3	0.5	2b	2.0	21	1.1	23	0.3	26	0.1	31
3	0.5	3b	2.4	21	0.7	23	0.4	26	0.3	31
3	0.8	1b	0.1	20	0.0	20	0.0	21	0.0	21
3	0.8	2b	0.2	20	0.0	20	0.0	21	0.0	21
3	0.8	3b	0.1	20	0.1	20	0.0	21	0.0	21

Hyp Set 1: ${H}_{0}:{\mu }_{1}={\mu }_{2}$ and ${H}_{1}:{\mu }_{1}<{\mu }_{2}$; Hyp Set 2: ${H}_{0}:{\mu }_{1}={\mu }_{2}$ and ${H}_{1}:{\mu }_{1}\ne {\mu }_{2}$

Hyp Set 3: ${H}_{1}:{\mu }_{1}>{\mu }_{2}$ and ${H}_{2}:{\mu }_{1}<{\mu }_{2}$. Underline: scenarios with one replication inconclusive

We first discuss how the error rate is influenced by the factors in the simulation study. The error rate is lower for the third hypotheses set than for the first and second. In other words, lowest error rates are observed when neither of the two hypotheses includes an equality constraint. For effect sizes $d>0$ the error rate of hypotheses set 1 is most often smaller than that of hypotheses set 2. In other words, a two sided alternative ${H}_{1}:{\mu }_{1}\ne {\mu }_{2}$ most often results in lower error rates than a one-sided alternative ${H}_{1}:{\mu }_{1}<{\mu }_{2}$. For effect sizes $d=0$ the error rates of hypotheses sets 1 and 2 are comparable.

There is a clear relation between the effect size and error rate. For effect sizes $d>0$ the error rate decreases with increasing effect size. Larger effect sizes are easier to capture and hence result in lower error rates. The error rates for $d=0$ are almost always below those for $d=0.2$ and quite often also below those for $d=0.5$, meaning the incorrect hypothesis is less often favoured when the data are generated under a zero effect size than when they are generated under a small or medium effect size.

For hypotheses sets 1 and 2 the error rate is also influenced by the fraction of the data that is used to specify the prior. The error rate decreases with increasing fraction when $d>0$. For larger fraction the variance of the prior increases and hence the complexity of ${H}_{0}$ increases. As a result of that ${BF}_{0u}$ decreases and ${BF}_{10}$ increases and there is a higher probability the correct hypothesis ${H}_{1}$ gets most support from the data. For the same reasoning the error rate increases with increasing fraction when $d=0$. Using more information from the data to specify the prior is advantageous for non-zero effect sizes but not for a zero effect size. For hypotheses set 3 the error rate is hardly influenced by the fraction, because ${BF}_{iu}$ does not depend on the fraction if the two hypotheses under consideration do not include an equality constraint [20].

Finally, Table 4 shows that the error rate decreases when ${BF}_{target}$ increases. For $d=0$.5 or 0.8 it decreases to (near) zero, while for $d=0$ or 0.2 it decreases to somewhat larger values. For large ${BF}_{target}$ strong support for a hypothesis is sought, hence it is unlikely that the incorrect hypothesis is favoured.

We now discuss how the mean group size is influenced by the factors in the simulation study. In almost all cases the mean group size is smaller for hypotheses set 3 than for hypotheses sets 1 and 2, so lower group sizes are needed when neither of the two hypotheses in a set includes an equality constraint. The mean group sizes for hypotheses sets 1 and 2 are comparable to one another.

The mean group size generally decreases when the effect size increases from $d=0.2$ to 0.5 and then further to 0.8. This is obvious since larger effect sizes are easier to capture and hence require a smaller sample. The mean group size for $d=0$ is most often below that for $d=0.2$ and for some scenarios even below that for $d=0.5.$

For hypotheses sets 1 and 2 the mean group size is also influenced by the fraction in the data to specify the prior. For $d=0$ it increases with increasing fraction. For other effect sizes this relation depends on the effect size and ${BF}_{target}$: sample size only increases with increasing fraction for combinations of low effect size and low ${BF}_{target}$. For hypotheses set 3 the mean group size is hardly influenced by the fraction.

Finally, the mean group size increases when ${BF}_{target}$ increases. As is obvious, larger group sizes are needed when a higher degree of support is required.

Tables 1-3 in the online supplement show how the error rate and mean group size change if the minimum group size decreases from 20 (Table S1) to 10 (Table S2) to 5 (Table S3). In most scenarios the error rate increases if the minimum group size becomes smaller. In other words, by chance due to starting with a small group size the incorrect hypothesis may be given more support by the data than the correct hypothesis. The main exceptions are those scenarios for hypotheses sets 1 and 2 with $d=0.2$. Furthermore, in almost all scenarios the mean group size decreases when a smaller minimum group size is used.

**Fig. 3:**
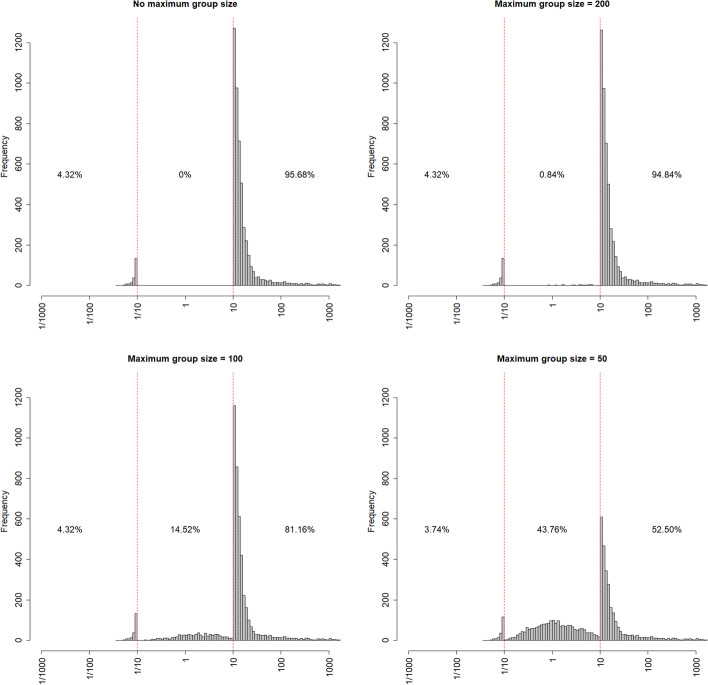
The effect of decreasing the maximum group size on the distribution of the Bayes factor

When the group size is limited to a certain maximum, there is a chance the result is inconclusive. This is illustrated in Fig. 3, which shows the distribution of $BF$ when there is no restriction on the group size, and when it is limited to 200, 100 or 50. This figure is based on the t-test for the first hypotheses set (${H}_{0}:{\mu }_{1}={\mu }_{2}$ versus ${H}_{1}:{\mu }_{1}<{\mu }_{2}$), an effect size $d=0.5$, a fraction $1b$, ${BF}_{target}=10$ and minimum group size ${N}_{min}=20$. The boundaries are represented by the two vertical dashed red lines. The percentages on top of each panel are the percentages for which ${BF<1/BF}_{target}$ (left), ${1/BF}_{target}<BF<{BF}_{target}$ (middle) and $BF>{BF}_{target}$ (right). In the case the group size is not limited, the incorrect hypothesis ${H}_{0}:{\mu }_{1}={\mu }_{2}$ is favoured in 4.32% if the cases and the correct hypothesis ${H}_{1}:{\mu }_{1}<{\mu }_{2}$ is favoured in 95.68% of the cases. When a maximum group size is used, some of the generated trials show an inconclusive result. When the maximum group size becomes smaller the percentage of such trails becomes larger, whereas the percentage trials for which the correct hypothesis is favoured becomes smaller. In general, such inconclusive results are more likely to occur when ${BF}_{target}$ increases and/or when the effect size decreases from 0.8 to 0.2.

It is to be expected that we that we observe decreasing percentage of indecisive results for growing sample sizes. This is mainly due to the consistency of the BF, which guarantees that for large enough sample sizes the Bayes factor ${BF}_{i{i}^{\text{'}}}$ converges to zero if ${H}_{{i}^{\text{'}}}$ is true or to infinity if ${H}_{i}$ is true. The four plots show that whenever we sample long enough the consistency will guarantee that we arrive at a conclusive result which passes any necessary threshold. However, the sample sizes required to attain such a threshold may, of course, be prohibitively large for practical research.

Tables 4-6 in the online supplement present error rates and means group sizes for the unequal variances t-test (i.e. Welch’s test). These are very similar to those of the equal variances t-test and in general the findings as discussed above for the equal variances t-test also hold for the Welch’s test.

## Conclusions and discussion

This paper introduced Bayesian updating to researchers in the biomedical field and showed results of a simulation study that investigated sample size and error rate. The results of the simulation study are intuitively sound and some of them are similar to those from a power analysis in the framework of null hypothesis significance testing. Larger sample size is needed when power increases just as a larger sample size is needed when ${BF}_{target}$ increases. Larger sample size is needed if the effect size decreases, whenever a power analysis is performed or Bayesian updating is used.

The results replicate those of previous simulation studies on Bayesian updating for two-group comparisons and confirm theoretical consistency results of the Bayes Factor under optional stopping: error rates and mean sample size decrease with increasing effect size, sample size increases with increasing ${BF}_{target}$ and error rate decreases with increasing ${BF}_{target}$ [17, 29]. The simulation study of this paper was more extensive since it used more than one set of hypotheses, allowed for unequal group variances and studied the effect of minimum and maximum group sizes. Another important difference is the choice of the prior. This study used part of the data to calculate the prior, while previous studies [17, 18] used the Jeffreys-Zellner-Siow prior ([30], implemented in the R package BayesFactor).

A simulation study with a wide range of factors and factor levels was used to study error rate and sample size. The R syntax on https://github.com/MirjamMoerbeek/BayesianUpdating can be used for other scenarios, for instance other effect sizes, other variances in both groups for Welch’s test, or a larger ${BF}_{target}$. As any simulation study, this one also had its limitations: it restricted to two-group comparisons, quantitative outcomes, a between-subject design and it did not take into account multilevel data structures, as may be encountered in cluster randomized trials. A focus on more complicated designs and other types of outcome variables is therefore necessary in future research. In addition to that, it might also be of interest to study the behaviour of the Bayes factor under model misspecification.

Bayesian updating is a viable alternative to a priori sample size calculations in studies where additional subjects can be recruited easily and data become available within a limited amount of time. It may not be applicable in longitudinal studies where the time between recruiting and measuring subjects is large. Also, there is a risk sufficient support for either hypothesis cannot be found since the sample size is limited, which may be the case in populations where a rare disease or disorder is studied. However, in such cases it is also very likely the sample size as obtained from an a priori sample size calculation exceeds the size of the population.

I hope readers of this paper will consider Bayesian updating an alternative to a priori sample size calculation, in experimental research as well as in observational research. The results in this paper inform them what sample size may be expected and how large the error rate is. These may be used in designing future studies for two-group comparisons.

## Supplementary Information

**Additional file 1: Table 1.** Percentage error and mean sample size for the equal variances t test $\left( {N_{{min}} = 20,N_{{max}} = 50000} \right)$. **Table 2.** Percentage error and mean sample size for the equal variances t test $\left( {N_{{min}} = 10,N_{{max}} = 50000} \right)$. **Table 3.** Percentage error and mean sample size for the equal variances t test $\left( {N_{{min}} = 5,N_{{max}} = 50000} \right)$. **Table 4.** Percentage error and mean sample size for the unequal variances t test (i.e. Welch’s test) $\left( {N_{{min}} = 20,N_{{max}} = 50000} \right)$. **Table 5.** Percentage error and mean sample size for the unequal variances t test (i.e. Welch’s test) $\left( {N_{{min}} = 10,N_{{max}} = 50000} \right)$. **Table 6.** Percentage error and mean sample size for the unequal variances t test (i.e. Welch’s test) $\left( {N_{{min}} = 5,N_{{max}} = 50000} \right)$.

## Data Availability

The Framingham dataset analysed during the current study is publicly available in the Data and Story repository, https://dasl.datadescription.com/datafile/framingham/?_sf_s=framingham&_sfm_cases=4+59943 R syntax to analyse these data is available on https://github.com/MirjamMoerbeek/BayesianUpdating This research uses on a simulation study. R syntax can be obtained from https://github.com/MirjamMoerbeek/BayesianUpdating

## References

[1] Cohen J (1988). Statistical power analysis for the behavioral sciences.

[2] Cohen J (1992). A power primer. Psychol Bull..

[3] Faul F, Erdfelder E, Lang A-G, Buchner A (2007). G*Power 3: A flexible statistical power analysis for the social, behavioral, and biomedical sciences. Behav Res Methods..

[4] Mayr S, Erdfelder E, Buchner A, Faul F (2007). A short tutorial of G*Power. Tutor Quant Methods Psychol..

[5] Statistical Solutions Ltd. (2017). nQuery. Sample size and power calculation.

[6] NCSS (2020). PASS 2020 power analysis and sample size software [Internet].

[7] Stein AC (1945). A two-sample test for a linear hypothesis whose power is independent of the variance. Ann Math Stat..

[8] Wittes J, Brittain E (1990). The role of internal pilot studies in increasing the efficiency of clinical trials. Stat Med..

[9] Wittes J, Schabenberger O, Zucker D, Brittain E, Proschan M (1999). Internal pilot studies I: type I error rate of the naive t-test. Stat Med..

[10] Jennison C, Turnbull BW (2000). Group sequential methods with applications to clinical trials.

[11] Wassmer G, Brannath W (2016). Group sequential and confirmatory adaptive designs in clinical trials.

[12] Gigerenzer G (2004). Mindless statistics. J Socio Econ..

[13] Kass RE, Raftery AE (1995). Bayes factors. J Am Stat Assoc..

[14] Jeffreys H (1961). Theory of probability.

[15] Fu Q, Hoijtink H, Moerbeek M (2021). Sample-size determination for the Bayesian t test and Welch ’ s test using the approximate adjusted fractional Bayes factor. Behav Res Methods..

[16] Hoijtink H, Mulder J, van Lissa C, Gu X (2019). A tutorial on testing hypotheses using the Bayes factor. Psychol Methods..

[17] Schönbrodt FD, Wagenmakers EJ, Zehetleitner M, Perugini M (2017). Sequential hypothesis testing with Bayes factors: efficiently testing mean differences. Psychol Methods..

[18] Stefan A, Gronau QF, Schönbrodt F, Wagenmakers E-J. A tutorial on Bayes factor design analysis using an informed prior. Behav Res Methods. 2017;51:1042–58. 10.3758/s13428-018-01189-8 PMC6538819 30719688

[19] Rouder JN (2014). Optional stopping: no problem for Bayesians. Psychon Bull Rev..

[20] Gu X, Mulder J, Hoijtink H (2018). Approximated adjusted fractional Bayes factors: A general method for testing informative hypotheses. Br J Math Stat Psychol..

[21] Hoijtink H, Gu X, Mulder J (2019). Bayesian evaluation of informative hypotheses for multiple populations. Br J Math Stat Psychol..

[22] O’Hagan A (1995). Fractional Bayes factors for model comparison. J R Stat Soc Ser B..

[23] Mulder J (2014). Prior adjusted default Bayes factors for testing (in) equality constrained hypotheses. Comput Stat Data Anal..

[24] Berger JO, PericchiL R (2004). Training samples in objective Bayesian model selection. Ann Stat..

[25] Berger JO, PericchiL R (1996). The intrinsic Bayes factor for model selection and prediction. J Am Stat Assoc..

[26] Kelter R (2020). Bayesian alternatives to null hypothesis significance testing in biomedical research: a non-technical introduction to Bayesian inference with JASP. BMC Med Res Methodol..

[27] Kahn HA, Sempos CT (1989). Statistical methods in epidemiology.

[28] R Core Team (2020). R: A language and environment for statistical computing.

[29] Hendriksen A, de Heide R, Grünwald P. Optional stopping with bayes factors: a categorization and extension of folklore results, with an application to invariant situations. arXiv. 2018;1–29.

[30] Rouder JN, Speckman PL, Sun D, Morey RD, Iverson G (2009). Bayesian t tests for accepting and rejecting the null hypothesis. Psychon Bull Rev..

