# Supplementary material for: Bayesian updating: increasing sample size during the course of a study
Source: BMC Med Res Methodol. 2021 Jul 5;21:137. doi: 10.1186/s12874-021-01334-6 (PMC8258966; doi:10.1186/s12874-021-01334-6)
Supplement: Supplementary file 1 — Additional file 1: Table 1. Percentage error and mean sample size for the equal variances t test $\left( {N_{{min}} = 20,N_{{max}} = 50000} \right)$Nmin=20,Nmax=50000. Table 2. Percentage error and mean sample size for the equal variances t test $\left( {N_{{min}} = 10,N_{{max}} = 50000} \right)$Nmin=10,Nmax=50000. Table 3. Percentage error and mean sample size for the equal variances t test $\left( {N_{{min}} = 5,N_{{max}} = 50000} \right)$Nmin=5,Nmax=50000. Table 4. Percentage error and mean sample size for the unequal variances t test (i.e. Welch’s test) $\left( {N_{{min}} = 20,N_{{max}} = 50000} \right)$Nmin=20,Nmax=50000. Table 5. Percentage error and mean sample size for the unequal variances t test (i.e. Welch’s test) $\left( {N_{{min}} = 10,N_{{max}} = 50000} \right)$ [file 12874_2021_1334_MOESM1_ESM.docx]

Bayesian updating: increasing sample size during the course of a study.

Supplementary Material

Mirjam Moerbeek

Utrecht University, the Netherlands

Correspondence concerning this article should be addressed to: Mirjam Moerbeek, Department of Methodology and Statistics, Utrecht University, PO Box 80140, 3508 TC Utrecht, The Netherlands. Phone: +31-(0)30-2531450. E-mail: m.moerbeek@uu.nl

Table 1. Percentage error and mean sample size for the equal variances t test ($N_{min}=20, N_{max}=50000)$.

|  |  |  |  | |  | |  | |  | |
| --- | --- | --- | --- | --- | --- | --- | --- | --- | --- | --- |
|  |  |  | BF_target_ = 3 | | BF_target_ = 5 | | BF_target_ = 10 | | BF_target_ = 20 | |
| HypSet | ES | fraction | % error | mean N | % error | mean N | % error | mean N | % error | mean N |
| 1 | 0 | 1b | 5.1 | 22 | 4.1 | 29 | 2.5 | 70 | 1.5 | 296 |
| 1 | 0 | 2b | 7.2 | 24 | 5.7 | 36 | 3.7 | 123 | 2.3 | 523 |
| 1 | 0 | 3b | 9.1 | 26 | 7.1 | 47 | 4.4 | 179 | 3.1 | 799 |
| 1 | 0.2 | 1b | 78.9 | 25 | 72.1 | 49 | 47.0 | 171 | 13.6 | 406 |
| 1 | 0.2 | 2b | 70.8 | 29 | 61.3 | 69 | 27.3 | 238 | 3.6 | 451 |
| 1 | 0.2 | 3b | 65.2 | 32 | 51.3 | 79 | 19.0 | 268 | 1.5 | 435 |
| 1 | 0.5 | 1b | 35.0 | 26 | 19.9 | 37 | 4.3 | 58 | 0.2 | 74 |
| 1 | 0.5 | 2b | 24.7 | 27 | 10.7 | 39 | 0.8 | 56 | 0.0 | 68 |
| 1 | 0.5 | 3b | 17.9 | 28 | 6.5 | 40 | 0.2 | 54 | 0.0 | 63 |
| 1 | 0.8 | 1b | 6.1 | 22 | 1.9 | 25 | 0.1 | 28 | 0.0 | 31 |
| 1 | 0.8 | 2b | 3.2 | 22 | 1.0 | 24 | 0.0 | 27 | 0.0 | 30 |
| 1 | 0.8 | 3b | 1.8 | 22 | 0.5 | 24 | 0.0 | 26 | 0.0 | 29 |
|  |  |  |  |  |  |  |  |  |  |  |
| 2 | 0 | 1b | 5.0 | 22 | 3.9 | 27 | 3.8 | 92 | 2.1 | 380 |
| 2 | 0 | 2b | 7.4 | 23 | 6.3 | 42 | 4.2 | 180 | 2.8 | 758 |
| 2 | 0 | 3b | 9.8 | 26 | 7.7 | 62 | 5.3 | 265 | 3.5 | 1114 |
| 2 | 0.2 | 1b | 88.3 | 23 | 84.7 | 34 | 56.6 | 170 | 10.1 | 512 |
| 2 | 0.2 | 2b | 82.4 | 25 | 73.3 | 56 | 30.0 | 282 | 1.1 | 543 |
| 2 | 0.2 | 3b | 78.9 | 28 | 62.2 | 83 | 17.2 | 342 | 0.0 | 531 |
| 2 | 0.5 | 1b | 50.5 | 24 | 34.7 | 35 | 2.5 | 70 | 0.0 | 85 |
| 2 | 0.5 | 2b | 39.8 | 26 | 15.4 | 44 | 0.1 | 67 | 0.0 | 78 |
| 2 | 0.5 | 3b | 31.9 | 28 | 6.3 | 48 | 0.0 | 64 | 0.0 | 75 |
| 2 | 0.8 | 1b | 13.9 | 23 | 4.7 | 26 | 0.0 | 31 | 0.0 | 34 |
| 2 | 0.8 | 2b | 7.7 | 23 | 1.1 | 26 | 0.0 | 29 | 0.0 | 33 |
| 2 | 0.8 | 3b | 5.6 | 23 | 0.1 | 26 | 0.0 | 29 | 0.0 | 32 |
|  |  |  |  |  |  |  |  |  |  |  |
| 3 | 0.2 | 1b | 19.2 | 26 | 12.8 | 41 | 7.0 | 71 | 3.8 | 114 |
| 3 | 0.2 | 2b | 18.5 | 26 | 13.1 | 40 | 7.3 | 71 | 3.3 | 114 |
| 3 | 0.2 | 3b | 19.8 | 26 | 13.3 | 39 | 7.4 | 70 | 3.4 | 113 |
| 3 | 0.5 | 1b | 2.1 | 21 | 0.8 | 23 | 0.3 | 26 | 0.2 | 31 |
| 3 | 0.5 | 2b | 2.0 | 21 | 1.1 | 23 | 0.3 | 26 | 0.1 | 31 |
| 3 | 0.5 | 3b | 2.4 | 21 | 0.7 | 23 | 0.4 | 26 | 0.3 | 31 |
| 3 | 0.8 | 1b | 0.1 | 20 | 0.0 | 20 | 0.0 | 21 | 0.0 | 21 |
| 3 | 0.8 | 2b | 0.2 | 20 | 0.0 | 20 | 0.0 | 21 | 0.0 | 21 |
| 3 | 0.8 | 3b | 0.1 | 20 | 0.1 | 20 | 0.0 | 21 | 0.0 | 21 |

Hyp Set 1: $H_{0}:\mu_{1}=\mu_{2}$ and $H_{1}:\mu_{1}<\mu_{2}$; Hyp Set 2: $H_{0}:\mu_{1}=\mu_{2}$ and $H_{1}:\mu_{1}\neq\mu_{2}$

Hyp Set 3: $H_{1}:\mu_{1}>\mu_{2}$ and $H_{2}:\mu_{1}<\mu_{2}$. Underline: scenarios with one replication inconclusive.

Table 2. Percentage error and mean sample size for the equal variances t test ($N_{min}=10, N_{max}=50000)$.

|  |  |  |  | |  | |  | |  | |
| --- | --- | --- | --- | --- | --- | --- | --- | --- | --- | --- |
|  |  |  | BF_target_ = 3 | | BF_target_ = 5 | | BF_target_ = 10 | | BF_target_ = 20 | |
| HypSet | ES | fraction | % error | mean N | % error | mean N | % error | mean N | % error | mean N |
| 1 | 0 | 1b | 7.6 | 12 | 6.0 | 19 | 4.5 | 64 | 2.1 | 268 |
| 1 | 0 | 2b | 10.7 | 14 | 9.0 | 29 | 5.8 | 121 | 3.9 | 542 |
| 1 | 0 | 3b | 13.6 | 16 | 10.3 | 38 | 7.4 | 173 | 4.1 | 783 |
| 1 | 0.2 | 1b | 79.0 | 14 | 72.8 | 32 | 47.8 | 154 | 13.1 | 402 |
| 1 | 0.2 | 2b | 70.3 | 18 | 61.2 | 52 | 28.8 | 220 | 3.9 | 436 |
| 1 | 0.2 | 3b | 65.2 | 20 | 52.7 | 64 | 17.5 | 256 | 1.1 | 423 |
| 1 | 0.5 | 1b | 44.2 | 15 | 26.4 | 27 | 5.8 | 52 | 0.1 | 69 |
| 1 | 0.5 | 2b | 31.3 | 17 | 14.5 | 30 | 1.4 | 50 | 0.0 | 63 |
| 1 | 0.5 | 3b | 23.2 | 19 | 8.9 | 31 | 0.6 | 48 | 0.0 | 60 |
| 1 | 0.8 | 1b | 15.5 | 13 | 5.8 | 17 | 0.5 | 23 | 0.0 | 26 |
| 1 | 0.8 | 2b | 8.2 | 14 | 1.8 | 17 | 0.1 | 21 | 0.0 | 24 |
| 1 | 0.8 | 3b | 5.0 | 13 | 0.9 | 16 | 0.0 | 20 | 0.0 | 24 |
|  |  |  |  |  |  |  |  |  |  |  |
| 2 | 0 | 1b | 8.3 | 12 | 7.1 | 22 | 5.4 | 88 | 3.3 | 379 |
| 2 | 0 | 2b | 11.9 | 15 | 10.6 | 40 | 6.5 | 180 | 4.2 | 717 |
| 2 | 0 | 3b | 16.2 | 20 | 13.9 | 58 | 9.1 | 271 | 4.2 | 1071 |
| 2 | 0.2 | 1b | 86.4 | 12 | 83.9 | 26 | 56.3 | 159 | 9.2 | 501 |
| 2 | 0.2 | 2b | 80.4 | 16 | 70.1 | 54 | 30.0 | 271 | 0.9 | 535 |
| 2 | 0.2 | 3b | 72.9 | 21 | 60.7 | 77 | 16.4 | 334 | 0.2 | 516 |
| 2 | 0.5 | 1b | 61.2 | 14 | 37.9 | 27 | 2.9 | 67 | 0.0 | 80 |
| 2 | 0.5 | 2b | 46.8 | 17 | 14.7 | 39 | 0.0 | 63 | 0.0 | 75 |
| 2 | 0.5 | 3b | 31.8 | 21 | 6.8 | 43 | 0.0 | 60 | 0.0 | 72 |
| 2 | 0.8 | 1b | 27.9 | 13 | 8.4 | 20 | 0.0 | 26 | 0.0 | 30 |
| 2 | 0.8 | 2b | 16.4 | 14 | 0.9 | 21 | 0.0 | 25 | 0.0 | 28 |
| 2 | 0.8 | 3b | 6.9 | 16 | 0.2 | 20 | 0.0 | 24 | 0.0 | 27 |
|  |  |  |  |  |  |  |  |  |  |  |
| 3 | 0.2 | 1b | 26.2 | 14 | 18.8 | 26 | 11.3 | 55 | 6.9 | 99 |
| 3 | 0.2 | 2b | 25.6 | 15 | 19.7 | 27 | 11.6 | 56 | 6.1 | 97 |
| 3 | 0.2 | 3b | 25.9 | 15 | 19.4 | 26 | 10.4 | 56 | 6.7 | 102 |
| 3 | 0.5 | 1b | 6.6 | 12 | 3.8 | 14 | 1.7 | 18 | 0.8 | 25 |
| 3 | 0.5 | 2b | 6.4 | 12 | 4.0 | 14 | 1.8 | 19 | 0.6 | 24 |
| 3 | 0.5 | 3b | 7.0 | 12 | 4.1 | 14 | 1.9 | 19 | 0.8 | 24 |
| 3 | 0.8 | 1b | 0.9 | 11 | 0.5 | 11 | 0.3 | 12 | 0.1 | 14 |
| 3 | 0.8 | 2b | 1.1 | 11 | 0.6 | 11 | 0.2 | 12 | 0.0 | 14 |
| 3 | 0.8 | 3b | 1.1 | 11 | 0.3 | 11 | 0.2 | 12 | 0.1 | 14 |

Hyp Set 1: $H_{0}:\mu_{1}=\mu_{2}$ and $H_{1}:\mu_{1}<\mu_{2}$; Hyp Set 2: $H_{0}:\mu_{1}=\mu_{2}$ and $H_{1}:\mu_{1}\neq\mu_{2}$

Hyp Set 3: $H_{1}:\mu_{1}>\mu_{2}$ and $H_{2}:\mu_{1}<\mu_{2}$. Underline: scenarios with one replication inconclusive. Double underline: scenarios with two replications inconclusive.

Table 3. Percentage error and mean sample size for the equal variances t test ($N_{min}=5, N_{max}=50000)$.

|  |  |  |  | |  | |  | |  | |
| --- | --- | --- | --- | --- | --- | --- | --- | --- | --- | --- |
|  |  |  | BF_target_ = 3 | | BF_target_ = 5 | | BF_target_ = 10 | | BF_target_ = 20 | |
| HypSet | ES | fraction | % error | mean N | % error | mean N | % error | mean N | % error | mean N |
| 1 | 0 | 1b | 11.5 | 7 | 9.7 | 15 | 7.4 | 60 | 4.9 | 260 |
| 1 | 0 | 2b | 15.5 | 10 | 13.1 | 23 | 9.0 | 111 | 6.2 | 538 |
| 1 | 0 | 3b | 20.3 | 12 | 15.3 | 33 | 9.6 | 165 | 6.5 | 732 |
| 1 | 0.2 | 1b | 75.9 | 9 | 71.2 | 24 | 47.7 | 145 | 12.9 | 393 |
| 1 | 0.2 | 2b | 65.9 | 11 | 59.0 | 42 | 27.6 | 206 | 3.7 | 416 |
| 1 | 0.2 | 3b | 60.0 | 14 | 49.3 | 56 | 17.6 | 229 | 1.3 | 408 |
| 1 | 0.5 | 1b | 47.2 | 10 | 29.8 | 21 | 6.6 | 47 | 0.2 | 66 |
| 1 | 0.5 | 2b | 34.0 | 12 | 15.8 | 25 | 1.3 | 46 | 0.0 | 60 |
| 1 | 0.5 | 3b | 25.5 | 13 | 9.2 | 27 | 0.3 | 44 | 0.0 | 56 |
| 1 | 0.8 | 1b | 22.4 | 8 | 8.5 | 14 | 0.6 | 20 | 0.0 | 23 |
| 1 | 0.8 | 2b | 12.8 | 9 | 2.6 | 13 | 0.1 | 18 | 0.0 | 22 |
| 1 | 0.8 | 3b | 7.5 | 9 | 1.0 | 13 | 0.0 | 17 | 0.0 | 21 |
|  |  |  |  |  |  |  |  |  |  |  |
| 2 | 0 | 1b | 14.4 | 8 | 11.4 | 21 | 9.0 | 88 | 5.5 | 353 |
| 2 | 0 | 2b | 20.9 | 13 | 16.6 | 37 | 11.9 | 168 | 7.5 | 713 |
| 2 | 0 | 3b | 24.9 | 18 | 20.0 | 53 | 12.2 | 245 | 8.5 | 1022 |
| 2 | 0.2 | 1b | 82.8 | 8 | 78.3 | 26 | 54.1 | 159 | 9.2 | 505 |
| 2 | 0.2 | 2b | 71.9 | 14 | 66.0 | 49 | 28.4 | 262 | 0.7 | 515 |
| 2 | 0.2 | 3b | 64.4 | 19 | 56.2 | 71 | 15.9 | 306 | 0.2 | 503 |
| 2 | 0.5 | 1b | 62.9 | 9 | 36.1 | 25 | 2.5 | 61 | 0.0 | 79 |
| 2 | 0.5 | 2b | 42.2 | 14 | 13.8 | 35 | 0.1 | 59 | 0.0 | 71 |
| 2 | 0.5 | 3b | 29.9 | 18 | 5.6 | 39 | 0.0 | 55 | 0.0 | 66 |
| 2 | 0.8 | 1b | 35.5 | 8 | 8.3 | 17 | 0.0 | 24 | 0.0 | 28 |
| 2 | 0.8 | 2b | 14.0 | 12 | 0.9 | 18 | 0.0 | 22 | 0.0 | 26 |
| 2 | 0.8 | 3b | 5.8 | 12 | 0.2 | 17 | 0.0 | 21 | 0.0 | 25 |
|  |  |  |  |  |  |  |  |  |  |  |
| 3 | 0.2 | 1b | 30.2 | 8 | 25.6 | 16 | 16.0 | 45 | 9.7 | 85 |
| 3 | 0.2 | 2b | 31.9 | 8 | 25.9 | 17 | 17.3 | 43 | 10.3 | 86 |
| 3 | 0.2 | 3b | 33.0 | 8 | 24.6 | 17 | 15.3 | 43 | 10.1 | 85 |
| 3 | 0.5 | 1b | 12.3 | 7 | 8.2 | 10 | 4.3 | 14 | 2.5 | 20 |
| 3 | 0.5 | 2b | 13.1 | 7 | 8.8 | 9 | 4.3 | 14 | 2.4 | 21 |
| 3 | 0.5 | 3b | 12.3 | 7 | 8.7 | 9 | 4.7 | 14 | 2.3 | 20 |
| 3 | 0.8 | 1b | 4.3 | 6 | 2.3 | 7 | 0.9 | 8 | 0.5 | 11 |
| 3 | 0.8 | 2b | 4.5 | 6 | 2.3 | 7 | 1.2 | 8 | 0.5 | 10 |
| 3 | 0.8 | 3b | 4.4 | 6 | 2.3 | 7 | 0.8 | 8 | 0.6 | 11 |

Hyp Set 1: $H_{0}:\mu_{1}=\mu_{2}$ and $H_{1}:\mu_{1}<\mu_{2}$; Hyp Set 2: $H_{0}:\mu_{1}=\mu_{2}$ and $H_{1}:\mu_{1}\neq\mu_{2}$

Hyp Set 3: $H_{1}:\mu_{1}>\mu_{2}$ and $H_{2}:\mu_{1}<\mu_{2}$. Underline: scenarios with one replication inconclusive.

Table 4. Percentage error and mean sample size for the unequal variances t test (i.e. Welch’s test) ($N_{min}=20, N_{max}=50000)$.

|  |  |  |  | |  | |  | |  | |
| --- | --- | --- | --- | --- | --- | --- | --- | --- | --- | --- |
|  |  |  | BF_target_ = 3 | | BF_target_ = 5 | | BF_target_ = 10 | | BF_target_ = 20 | |
| HypSet | ES | fraction | % error | mean N | % error | mean N | % error | mean N | % error | mean N |
| 1 | 0 | 1b | 4.7 | 22 | 4.0 | 28 | 2.8 | 71 | 1.6 | 275 |
| 1 | 0 | 2b | 7.9 | 24 | 5.7 | 38 | 4.4 | 127 | 2.0 | 538 |
| 1 | 0 | 3b | 9.0 | 26 | 8.0 | 45 | 5.2 | 182 | 3.1 | 757 |
| 1 | 0.2 | 1b | 79.8 | 25 | 71.7 | 47 | 47.7 | 171 | 13.8 | 416 |
| 1 | 0.2 | 2b | 71.0 | 28 | 60.7 | 65 | 27.7 | 233 | 3.8 | 441 |
| 1 | 0.2 | 3b | 65.2 | 32 | 52.8 | 79 | 18.9 | 263 | 1.6 | 428 |
| 1 | 0.5 | 1b | 32.9 | 26 | 19.1 | 38 | 4.0 | 57 | 0.2 | 72 |
| 1 | 0.5 | 2b | 23.0 | 27 | 10.1 | 39 | 1.0 | 56 | 0.0 | 67 |
| 1 | 0.5 | 3b | 18.5 | 28 | 6.2 | 39 | 0.3 | 53 | 0.0 | 64 |
| 1 | 0.8 | 1b | 6.0 | 22 | 2.2 | 25 | 0.2 | 28 | 0.0 | 32 |
| 1 | 0.8 | 2b | 3.3 | 22 | 0.8 | 24 | 0.0 | 27 | 0.0 | 29 |
| 1 | 0.8 | 3b | 2.3 | 22 | 0.4 | 24 | 0.0 | 26 | 0.0 | 29 |
|  |  |  |  |  |  |  |  |  |  |  |
| 2 | 0 | 1b | 5.7 | 22 | 4.1 | 27 | 2.8 | 94 | 2.2 | 366 |
| 2 | 0 | 2b | 7.4 | 23 | 6.9 | 42 | 4.1 | 189 | 2.7 | 734 |
| 2 | 0 | 3b | 9.5 | 26 | 7.8 | 60 | 5.2 | 268 | 4.0 | 1061 |
| 2 | 0.2 | 1b | 88.5 | 22 | 85.4 | 33 | 55.5 | 174 | 9.9 | 507 |
| 2 | 0.2 | 2b | 81.9 | 25 | 73.7 | 57 | 30.5 | 280 | 1.0 | 551 |
| 2 | 0.2 | 3b | 78.1 | 28 | 62.4 | 82 | 15.9 | 336 | 0.1 | 526 |
| 2 | 0.5 | 1b | 50.4 | 25 | 32.9 | 36 | 2.7 | 70 | 0.0 | 84 |
| 2 | 0.5 | 2b | 39.8 | 26 | 15.7 | 44 | 0.1 | 67 | 0.0 | 78 |
| 2 | 0.5 | 3b | 30.4 | 28 | 6.7 | 48 | 0.0 | 64 | 0.0 | 75 |
| 2 | 0.8 | 1b | 14.0 | 23 | 5.3 | 26 | 0.0 | 31 | 0.0 | 34 |
| 2 | 0.8 | 2b | 7.6 | 23 | 1.1 | 26 | 0.0 | 29 | 0.0 | 32 |
| 2 | 0.8 | 3b | 5.6 | 23 | 0.2 | 26 | 0.0 | 29 | 0.0 | 31 |
|  |  |  |  |  |  |  |  |  |  |  |
| 3 | 0.2 | 1b | 18.9 | 27 | 13.1 | 40 | 6.9 | 71 | 3.6 | 111 |
| 3 | 0.2 | 2b | 20.2 | 26 | 13.4 | 40 | 6.7 | 73 | 3.1 | 113 |
| 3 | 0.2 | 3b | 18.8 | 26 | 13.6 | 40 | 7.2 | 70 | 3.6 | 114 |
| 3 | 0.5 | 1b | 1.7 | 21 | 1.2 | 23 | 0.3 | 26 | 0.1 | 31 |
| 3 | 0.5 | 2b | 2.2 | 21 | 1.0 | 23 | 0.4 | 26 | 0.2 | 31 |
| 3 | 0.5 | 3b | 2.0 | 21 | 0.7 | 23 | 0.6 | 26 | 0.2 | 30 |
| 3 | 0.8 | 1b | 0.1 | 20 | 0.1 | 20 | 0.0 | 21 | 0.0 | 22 |
| 3 | 0.8 | 2b | 0.2 | 20 | 0.0 | 20 | 0.0 | 21 | 0.0 | 21 |
| 3 | 0.8 | 3b | 0.1 | 20 | 0.0 | 20 | 0.1 | 21 | 0.0 | 22 |

Hyp Set 1: $H_{0}:\mu_{1}=\mu_{2}$ and $H_{1}:\mu_{1}<\mu_{2}$; Hyp Set 2: $H_{0}:\mu_{1}=\mu_{2}$ and $H_{1}:\mu_{1}\neq\mu_{2}$

Hyp Set 3: $H_{1}:\mu_{1}>\mu_{2}$ and $H_{2}:\mu_{1}<\mu_{2}$. Underline: scenarios with one replication inconclusive.

Table 5. Percentage error and mean sample size for the unequal variances t test (i.e. Welch’s test) ($N_{min}=10, N_{max}=50000)$.

|  |  |  |  | |  | |  | |  | |
| --- | --- | --- | --- | --- | --- | --- | --- | --- | --- | --- |
|  |  |  | BF_target_ = 3 | | BF_target_ = 5 | | BF_target_ = 10 | | BF_target_ = 20 | |
| HypSet | ES | fraction | % error | mean N | % error | mean N | % error | mean N | % error | mean N |
| 1 | 0 | 1b | 7.7 | 12 | 6.5 | 19 | 4.8 | 61 | 3.1 | 275 |
| 1 | 0 | 2b | 11.5 | 15 | 9.2 | 28 | 6.2 | 119 | 4.1 | 521 |
| 1 | 0 | 3b | 13.7 | 16 | 10.8 | 37 | 7.7 | 163 | 4.8 | 754 |
| 1 | 0.2 | 1b | 78.5 | 14 | 72.8 | 33 | 47.4 | 156 | 13.3 | 402 |
| 1 | 0.2 | 2b | 71.5 | 18 | 60.8 | 52 | 29.6 | 209 | 3.8 | 433 |
| 1 | 0.2 | 3b | 63.8 | 20 | 51.5 | 64 | 18.5 | 252 | 1.4 | 426 |
| 1 | 0.5 | 1b | 44.4 | 15 | 26.4 | 27 | 5.6 | 52 | 0.2 | 69 |
| 1 | 0.5 | 2b | 31.6 | 17 | 14.5 | 30 | 1.6 | 50 | 0.0 | 62 |
| 1 | 0.5 | 3b | 23.8 | 19 | 8.6 | 31 | 0.5 | 48 | 0.0 | 59 |
| 1 | 0.8 | 1b | 14.5 | 13 | 6.1 | 18 | 0.5 | 23 | 0.0 | 26 |
| 1 | 0.8 | 2b | 8.4 | 13 | 2.3 | 17 | 0.0 | 21 | 0.0 | 25 |
| 1 | 0.8 | 3b | 5.5 | 14 | 0.8 | 16 | 0.0 | 20 | 0.0 | 23 |
|  |  |  |  |  |  |  |  |  |  |  |
| 2 | 0 | 1b | 8.1 | 12 | 7.2 | 22 | 5.1 | 91 | 3.2 | 367 |
| 2 | 0 | 2b | 12.4 | 15 | 10.5 | 40 | 7.0 | 176 | 4.5 | 742 |
| 2 | 0 | 3b | 15.6 | 20 | 13.2 | 58 | 8.3 | 262 | 4.9 | 1076 |
| 2 | 0.2 | 1b | 86.5 | 12 | 82.5 | 26 | 56.7 | 162 | 9.4 | 499 |
| 2 | 0.2 | 2b | 80.0 | 16 | 69.4 | 53 | 30.7 | 270 | 0.9 | 531 |
| 2 | 0.2 | 3b | 73.0 | 22 | 59.5 | 77 | 16.6 | 322 | 0.2 | 522 |
| 2 | 0.5 | 1b | 61.0 | 13 | 37.9 | 28 | 2.8 | 66 | 0.0 | 81 |
| 2 | 0.5 | 2b | 46.3 | 17 | 14.9 | 39 | 0.0 | 63 | 0.0 | 76 |
| 2 | 0.5 | 3b | 31.8 | 21 | 5.9 | 43 | 0.0 | 59 | 0.0 | 71 |
| 2 | 0.8 | 1b | 29.0 | 13 | 9.0 | 19 | 0.0 | 26 | 0.0 | 30 |
| 2 | 0.8 | 2b | 15.1 | 15 | 1.0 | 20 | 0.0 | 24 | 0.0 | 29 |
| 2 | 0.8 | 3b | 6.7 | 15 | 0.1 | 20 | 0.0 | 23 | 0.0 | 27 |
|  |  |  |  |  |  |  |  |  |  |  |
| 3 | 0.2 | 1b | 26.4 | 15 | 20.2 | 26 | 10.7 | 59 | 6.2 | 97 |
| 3 | 0.2 | 2b | 25.5 | 15 | 19.5 | 27 | 11.4 | 55 | 6.3 | 100 |
| 3 | 0.2 | 3b | 25.2 | 15 | 19.2 | 25 | 11.3 | 55 | 5.9 | 102 |
| 3 | 0.5 | 1b | 6.9 | 12 | 4.3 | 14 | 1.9 | 18 | 0.8 | 24 |
| 3 | 0.5 | 2b | 6.8 | 12 | 3.7 | 14 | 1.8 | 18 | 1.0 | 24 |
| 3 | 0.5 | 3b | 6.7 | 12 | 4.1 | 14 | 1.4 | 19 | 0.8 | 24 |
| 3 | 0.8 | 1b | 0.9 | 11 | 0.5 | 11 | 0.2 | 12 | 0.1 | 14 |
| 3 | 0.8 | 2b | 0.9 | 11 | 0.6 | 11 | 0.2 | 12 | 0.1 | 14 |
| 3 | 0.8 | 3b | 1.4 | 10 | 0.5 | 11 | 0.2 | 12 | 0.1 | 14 |

Hyp Set 1: $H_{0}:\mu_{1}=\mu_{2}$ and $H_{1}:\mu_{1}<\mu_{2}$; Hyp Set 2: $H_{0}:\mu_{1}=\mu_{2}$ and $H_{1}:\mu_{1}\neq\mu_{2}$

Hyp Set 3: $H_{1}:\mu_{1}>\mu_{2}$ and $H_{2}:\mu_{1}<\mu_{2}$. Underline: scenarios with one replication inconclusive.

Table 6. Percentage error and mean sample size for the unequal variances t test (i.e. Welch’s test) ($N_{min}=5, N_{max}=50000)$.

|  |  |  |  | |  | |  | |  | |
| --- | --- | --- | --- | --- | --- | --- | --- | --- | --- | --- |
|  |  |  | BF_target_ = 3 | | BF_target_ = 5 | | BF_target_ = 10 | | BF_target_ = 20 | |
| HypSet | ES | fraction | % error | mean N | % error | mean N | % error | mean N | % error | mean N |
| 1 | 0 | 1b | 11.7 | 7 | 9.6 | 15 | 7.4 | 60 | 4.5 | 277 |
| 1 | 0 | 2b | 16.7 | 9 | 13.9 | 24 | 9.1 | 116 | 6.8 | 484 |
| 1 | 0 | 3b | 20.1 | 11 | 16.0 | 31 | 11.5 | 174 | 6.9 | 692 |
| 1 | 0.2 | 1b | 76.4 | 9 | 71.5 | 25 | 47.7 | 141 | 13.0 | 395 |
| 1 | 0.2 | 2b | 66.5 | 12 | 57.3 | 44 | 28.7 | 199 | 3.7 | 416 |
| 1 | 0.2 | 3b | 60.4 | 14 | 48.7 | 56 | 17.7 | 233 | 1.7 | 413 |
| 1 | 0.5 | 1b | 48.0 | 10 | 30.1 | 21 | 6.6 | 46 | 0.2 | 65 |
| 1 | 0.5 | 2b | 34.5 | 12 | 16.1 | 25 | 1.6 | 46 | 0.0 | 60 |
| 1 | 0.5 | 3b | 25.6 | 13 | 10.3 | 27 | 0.3 | 43 | 0.0 | 55 |
| 1 | 0.8 | 1b | 22.4 | 9 | 9.0 | 14 | 0.7 | 20 | 0.0 | 24 |
| 1 | 0.8 | 2b | 12.3 | 9 | 3.0 | 13 | 0.1 | 18 | 0.0 | 21 |
| 1 | 0.8 | 3b | 7.8 | 9 | 1.6 | 13 | 0.0 | 17 | 0.0 | 21 |
|  |  |  |  |  |  |  |  |  |  |  |
| 2 | 0 | 1b | 13.9 | 8 | 12.9 | 21 | 8.5 | 84 | 6.0 | 350 |
| 2 | 0 | 2b | 21.5 | 13 | 16.4 | 37 | 11.6 | 168 | 8.1 | 705 |
| 2 | 0 | 3b | 25.5 | 18 | 19.0 | 55 | 13.6 | 246 | 8.9 | 1015 |
| 2 | 0.2 | 1b | 81.4 | 8 | 77.6 | 25 | 52.9 | 156 | 9.6 | 478 |
| 2 | 0.2 | 2b | 72.4 | 14 | 65.1 | 48 | 27.2 | 261 | 1.0 | 507 |
| 2 | 0.2 | 3b | 65.3 | 19 | 54.2 | 71 | 14.8 | 315 | 0.1 | 498 |
| 2 | 0.5 | 1b | 62.1 | 9 | 36.0 | 25 | 2.5 | 60 | 0.0 | 77 |
| 2 | 0.5 | 2b | 42.8 | 14 | 14.9 | 35 | 0.0 | 59 | 0.0 | 70 |
| 2 | 0.5 | 3b | 28.5 | 18 | 5.6 | 38 | 0.0 | 56 | 0.0 | 66 |
| 2 | 0.8 | 1b | 35.7 | 9 | 8.5 | 17 | 0.0 | 24 | 0.0 | 28 |
| 2 | 0.8 | 2b | 13.8 | 12 | 0.7 | 17 | 0.0 | 22 | 0.0 | 26 |
| 2 | 0.8 | 3b | 6.3 | 12 | 0.1 | 16 | 0.0 | 21 | 0.0 | 25 |
|  |  |  |  |  |  |  |  |  |  |  |
| 3 | 0.2 | 1b | 32.3 | 8 | 25.6 | 17 | 16.4 | 42 | 9.5 | 85 |
| 3 | 0.2 | 2b | 32.1 | 8 | 24.7 | 17 | 15.6 | 44 | 10.0 | 85 |
| 3 | 0.2 | 3b | 31.4 | 8 | 25.2 | 17 | 17.9 | 42 | 10.1 | 84 |
| 3 | 0.5 | 1b | 13.3 | 7 | 8.7 | 9 | 5.1 | 14 | 2.6 | 20 |
| 3 | 0.5 | 2b | 12.4 | 7 | 8.8 | 9 | 4.6 | 14 | 2.9 | 20 |
| 3 | 0.5 | 3b | 13.0 | 7 | 8.3 | 9 | 4.8 | 14 | 2.8 | 20 |
| 3 | 0.8 | 1b | 4.5 | 6 | 2.0 | 7 | 1.0 | 8 | 0.5 | 10 |
| 3 | 0.8 | 2b | 4.6 | 6 | 2.5 | 7 | 1.5 | 8 | 0.3 | 11 |
| 3 | 0.8 | 3b | 4.8 | 6 | 2.4 | 7 | 0.9 | 9 | 0.6 | 11 |

Hyp Set 1: $H_{0}:\mu_{1}=\mu_{2}$ and $H_{1}:\mu_{1}<\mu_{2}$; Hyp Set 2: $H_{0}:\mu_{1}=\mu_{2}$ and $H_{1}:\mu_{1}\neq\mu_{2}$

Hyp Set 3: $H_{1}:\mu_{1}>\mu_{2}$ and $H_{2}:\mu_{1}<\mu_{2}$. Double underline: scenarios with two replications inconclusive.
